# The association between rural/urban residence status and patient-reported outcomes in individuals with Chronic Obstructive Pulmonary Disease (COPD): Protocol for a systematic review and meta-analysis

**DOI:** 10.1371/journal.pone.0340451

**Published:** 2026-01-06

**Authors:** McKenzie Granata Green, Laurie L. Meschke, Thankam Sunil, Javiette Samuel, Kristina W. Kintziger, Phoebe M. Tran

**Affiliations:** 1 Department of Public Health, the University of Tennessee, Knoxville, Knoxville, Tennessee, United States of America; 2 Office of Community Engagement and Outreach, the University of Tennessee, Knoxville, Knoxville, Tennessee, United States of America; 3 College of Public Health, University of Nebraska Medical Center, Omaha Nebraska, United States of America; Faculty of Medicine Universitas Indonesia - CIPTO Mangunkusumo General Hospital, INDONESIA

## Abstract

**Introduction:**

Rural residency is associated with a disproportionate burden of chronic obstructive pulmonary disease (COPD) and poorer COPD health outcomes. While increasing focus has been placed on the influence of rural/urban residence on clinical outcomes, little is known about the impact of rural versus urban residency status on patient-reported outcome measures (PROMs) in individuals with COPD, despite the use of PROMs to tailor interventions and treatments to individual patient needs.

**Objective:**

The objective of this review is to synthesize evidence of a relation between rural/urban residency status and PROMs in individuals with COPD.

**Methods:**

Beginning May 2025, we will search EBSCO, Elsevier, Cochrane Library, PubMed, and relevant websites to identify research published between January 1, 2012, and November 1, 2024. Two reviewers will independently screen titles, abstracts, and full texts, with a third reviewer to resolve any discrepancies. All data sources and selection management will be fulfilled and housed in the Covidence systematic review software. The primary outcome of this review is the association between rural/urban residency and PROMs in individuals with COPD. If appropriate, a meta-analysis will be conducted. Sub-group analysis will be performed by sex. Sensitivity analysis will be performed by excluding studies with “low quality” based on risk of bias assessment.

**Ethics and dissemination:**

This study is exempt from institutional review as it will be a secondary analysis of published data. Results of this study are expected by September 2025 and will be disseminated in a relevant peer-reviewed journal.

**Trial registration:**

Prospero registration number: CRD42024627343.

## Introduction

Chronic obstructive pulmonary disease (COPD), a collection of lower respiratory diseases, is a leading cause of death and disability in the United States [1]. As of July 2024, approximately 16 million Americans live with COPD [2]. Among individuals with COPD, a disproportionate burden has been seen in those living in rural areas compared to their urban counterparts [3] with 8.2% of individuals living in rural America having a COPD diagnosis compared to 4.7% of individuals living in urban America [4]. Additionally, rural patients with COPD have acute exacerbations of COPD (AECOPD)-related hospitalization rates 21% higher than their urban counterparts [5]. Furthermore, rural patients with COPD experience greater obstacles to receiving care than their urban counterparts [3], such as higher rates of poverty (15.4% in rural areas compared to 11.9% in urban areas) [6] and reduced access to medical care due to a shortage of primary care and specialty physicians in rural areas [7,8].

The diverse range of symptoms that accompany COPD heavily influence a patient's health and clinical outcomes. As such, healthcare providers use validated tools to assess patient health status and quality of life [9]. Such tools include patient-reported outcome measures, or PROMs, which consist of outcomes derived directly from the patient regarding the status of their health [10]. Unlike other tools, PROMs are unmodified by clinicians, therefore directly reflecting the experiences of the patient [11]. Despite PROMs being associated with increases in healthcare quality and equity and the growing recognition of the importance of incorporating patient experiences into healthcare [12], few studies have examined associations between rural and urban residency status and PROMs experienced by individuals with COPD [13]. As rural residency has been shown to be a risk factor for COPD, even among never-smokers [14] and associated with higher all-cause mortality rates [15], synthesizing and critically evaluating the currently available literature in a systemic review will promote researcher and clinician understanding of how, if at all, residency status influences COPD patient-reported health outcomes. Likewise, findings from this systematic review can be used to support strategic implementation and allocation of pulmonary care resources.

### Objective

The purpose of this report is to describe the methodology that will be used to conduct a systematic review and meta-analysis that aims to answer the following research questions. 1) Does rural vs urban residence status impact PROMs of individuals with COPD in the United States? 2) Are there sex differences in the impact of rural vs urban residence status on PROMs in individuals with COPD in the United States?

## Methods

This review adheres to the Preferred Reporting Items for Systematic Reviews and Meta-Analysis Protocols (PRISMA-P) guidelines [16] for the protocol, systematic review, and meta-analysis. This protocol has been registered on the International Prospective Register of Systematic Reviews (PROSPERO) database (ID: CRD42024627343).

### Search strategy and informational sources

Beginning May 2025, a literature search using ESBCO (includes CINHAL and Health Policy References Centers), Elsevier (includes Embase, Science Direct, and Scopus), Cochrane Library, and PubMed, and relevant organization websites such as the American Lung Association (ALA) and Centers for Disease Control and Prevention (CDC) will be conducted to identify publications published between January 1, 2012, and December 31, 2024. The participants, exposure, comparison, and outcome (PECO) criteria [17], as well as concepts and keywords in the search strategy for the systematic review can be found in Table 1. Each search strategy concept will use appropriate keywords, informed by previous search strategies and the University of Tennessee, Knoxville’s Health Sciences Librarian. The search strategy was developed for each database by the first author in consultation with the University of Tennessee's Health Sciences Librarian.

**Table 1:** Population Exposure Comparison Outcome (PECO) summary.

Element	MeSH	Keywords
**Population:** Adults (18+) living with COPD in the United States	chronic obstructive pulmonary disease, COPD, chronic bronchitis, emphysema, airway obstruction, pulmonology, pulmonary, BODE Index	COPD*
**Exposure:** Rural residency status	rural, rural residency, rural-urban continuum codes, RUC codes, nonmetro	Residency status* Rural*
**Comparison:**	rural vs urban residency	Urban*
**Outcome:** Patient-reported outcome measures	patient-reported outcome measures, PROMs, health-related quality of life, HRQL, functional status, symptoms and symptom burden, health behaviors, patient experience	PROMs*, HRQL*

### Eligibility criteria

**Fig 1:**
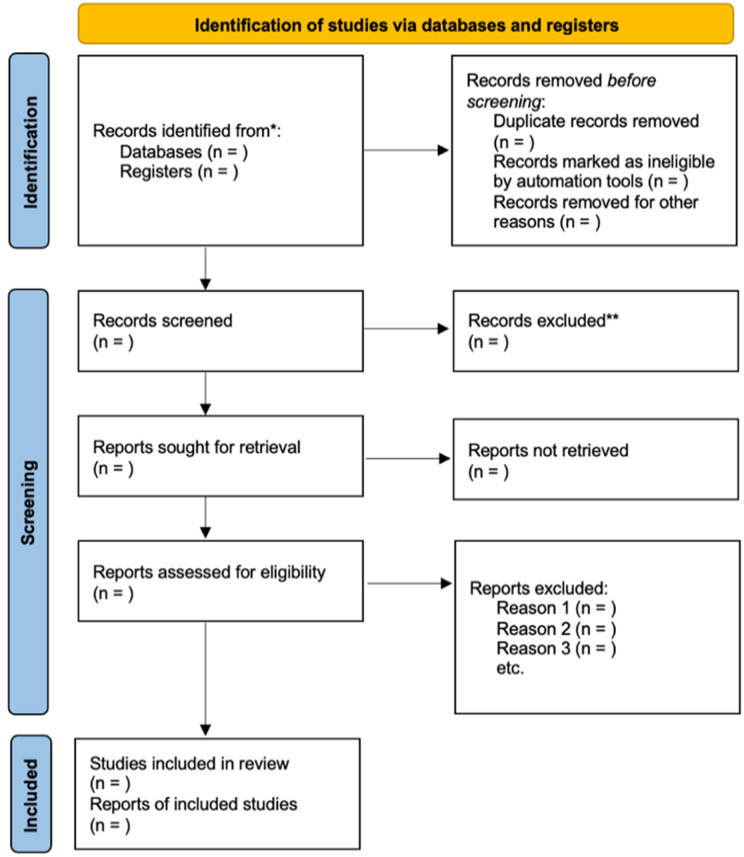
Flow chart of literature search and selection based on Prisma 2020 Flow Diagram [23].

As the past decade has shown a dramatic increase in the development of strategies for detecting COPD [18], studies included in this review will have been conducted between January 1, 2012 and December 31, 2024 to ensure study relevance and quality of evidence. We will include studies conducted in the United States (U.S.) or its territories as we are looking at COPD outcomes of U.S. residents. Furthermore, included studies will be limited to those published in English or Spanish. This decision was made based on a preliminary search of the literature in which 100% of relevant publications were conducted in English. However, as Spanish is the second most prominent language in the United States [19], we did not want to exclude any relevant articles that may be published in Spanish. Additionally, studies will contain individuals with COPD as diagnosed using American Lung Association-recognized diagnostic criteria (e.g., spirometry test, lung volume test, diffusing capacity test, etc.), and studies that examine one or more PROMs will be included in this systematic review. Furthermore, this review will include the following study designs: peer-reviewed randomized control trials (RCTs) and observational studies (cohort, case-control, cross-sectional, and ecological studies). Animal studies and case studies or case series will not be included. No restrictions will be placed on study settings. Only reports that can be downloaded or are available in print will be included in this review. A flow chart of the literature search and selection strategy can be seen in Fig 1.

### Data extraction and management

Beginning in May 2025, eligible reports will be imported and housed in the Covidence systematic review software [20]. After deduplication, record titles/abstracts will be independently screened by two reviewers a predefined inclusion criterion. Excluded reports will be documented with the rationale for exclusion. Any discrepancies between reviewers will be resolved by a third reviewer. We anticipate record screening to be complete by the end of July 2025.

After screening, reviewers will extract data using a standardized data extraction tool in Covidence. Extracted data will include information such as first author name, publication year, study design, study objectives/research questions, PROM used (i.e., health-related quality of life (HRQL), functional status, symptoms and symptom burden, health behaviors, or patient experiences), measure of association (odds ratio, risk difference, risk ratio, rate ratio), measure of variance, and rural/urban measure [21]. Extracted data will be prepared for analysis utilizing R statistical software [22]. We anticipate data extraction to be complete by the end of August 2025.

The primary outcome of this systematic review is to examine the relationship between rural and urban residence status and PROMs among individuals with COPD in the United States. Should there be any key missing information, including rural/urban residence status, PROM definition, or results of the analysis, we will attempt to contact study authors. Furthermore, forward citation chaining will be conducted to identify additional eligible studies. Results from this study will be generated by the end of September 2025.

### Quality assessment

The Cochrane Risk of Bias Tool 2.0 [24] will be used to assess the quality of experimental/randomized control studies included in this systematic review. The tool provides a framework for assessing the risk of bias for a single result by 1) specifying the result being assessed, 2) specifying the result of interest, 3) listing sources and information being assessed, 4) answering signaling questions, 5) judging the risk of bias in each domain, and 6) judging the overall risk of bias for the result [25].

The NIH Study Quality Assessment Tool for Observational Cohort and Cross-Sectional Studies and NIH Study Quality Assessment Tool for Case-control Studies [26] will be used to assess the quality of observational studies (Cohort/Cross-sectional and Case-control, respectively) included in this systematic review. While not standardized, these tools are specific to their respective study designs and have been used by subject matter experts in various medical science fields [26], such as biomedical engineering [27]. Additionally, these tools assess for potential bias by answering a series of questions (14 questions included in the cohort and cross-sectional tool and 12 questions included in the case-control tool) as yes, no, cannot determine, not applicable, or not reported [26].

### Statistical analyses

Primary studies included in this review may have significant heterogeneity in their research methods (e.g., study design, population characteristics, study setting, exposure and outcome measurement, length of follow-up, etc.). As such, it may be inappropriate to summarize and combine the included studies into one summary measurement with meta-analysis. Should, however, three or more studies report effect measures and 95% CIs for the same PROM, or if three or more different PROMs measure a specific construct, a meta-analysis will be conducted in the R package *metafor,* [28]. If a meta-analysis is warranted, effect sizes and ratio measures will be converted into standardized mean differences and combined using a random-effects model with inverse-variance weighting to calculate pooled effect sizes [10,29]. I^2^ statistics will be used to describe the percentage of variation across included studies [30], with I^2^ values of <25%, 25%−50%, 51%−75%, and >75% indicating low, moderate, medium, and high heterogeneity, respectively, based off the Cochrane handbook and prior literature [10,31].

### Sub-group and sensitivity analysis

Prior literature has shown that females with COPD experience greater dyspnea, or shortness of breath [32], and a higher morbidity compared to their male counterparts [33]. As such, a sub-group analysis will be performed by sex. Sensitivity analysis will be performed by excluding studies with “low quality” according to the appropriate risk of bias tool and comparing the results with the overall analysis [10,34].

### Addressing meta-biases

Biases will be assessed and addressed using appropriate statistical tests and methods. Biases to be aware of while conducting this review include, but are not limited to confounding bias, non-reporting bias, and publication bias.

Confounding bias due to potential confounding variables, such as income, insurance status, and provider status will be addressed qualitatively by describing the variables that were included in the primary studies as confounders and describing those that were potential confounders but not included.

Non-reporting bias [35] and publication bias [36] will be addressed using a funnel plot, with a symmetrical plot indicating a study is absent of bias and an asymmetrical plot indicating bias is present, and meta-regressions as funnel plots are frequently subjective and sensitive to sample size. Furthermore, heterogeneity in PROM measurement will be evaluated using the I^2^ statistic.

All identified biases and the procedures taken to mitigate them will be documented in the final systematic review with a detailed description of their potential impact on the review’s conclusion.

### Confidence in cumulative evidence

Confidence in the cumulative evidence will be assessed using the Grading of Recommendations Assessment, Development, and Evaluation (GRADE) approach, which defines evidence as high quality, moderate quality, low quality, and very low quality [37].

### Ethics and dissemination

As this study is a secondary analysis containing published data, this study is exempt from institutional review board approval [38]. No new participants will be included in this study. Completion of this study is expected in September of 2025. All data and findings from this review will be made available to the public upon completion of the study.

## Discussion

COPD is a leading cause of death and disability in the United States [3]. Among those with COPD, a disproportionate burden has been seen in those living in rural areas compared to their urban counterparts [12]. PROMs allow interventions and treatments to be tailored to individual needs [39], and their use in comprehensive assessments of pulmonary diseases in clinical and research settings has been shown to strengthen treatment and intervention outcomes [40]. While other fields of medicine have shown differences in PROMs in rural versus urban residents [41], there is little literature examining the differences in PROMs in rural/urban residents living with COPD. The available literature has yet to be systematically reviewed and evaluated. By quantifying the association between rural/urban resident status and PROMs experienced by individuals with COPD and identifying any male/female sex differences, such a review can provide valuable insights that may not be captured within primary studies, allowing practitioners to evaluate the effectiveness of treatments from the patients’ perspective [42] and health systems to assess outcomes and treatment efficiency [43] in rural/urban patients with COPD.

The proposed systematic review and meta-analysis will face several challenges including, but not limited to, a lack of available studies or studies with incomplete data, studies not being available in English, and high heterogeneity, as studies may contain a wide variety of PROMs and definitions of rural/urban residence. In the instance that there are too few studies for a systematic review, a scoping review will be conducted. To assess sources of heterogeneity, a meta-regression will be conducted.

## Supporting information

S1 Checklist PRISMA-P-checklist. (PDF)
